# Origins, actions and dynamic expression patterns of the neuropeptide VGF in rat peripheral and central sensory neurones following peripheral nerve injury

**DOI:** 10.1186/1744-8069-4-62

**Published:** 2008-12-10

**Authors:** Andrew Moss, Rachel Ingram, Stephanie Koch, Andria Theodorou, Lucie Low, Mark Baccei, Gareth J Hathway, Michael Costigan, Stephen R Salton, Maria Fitzgerald

**Affiliations:** 1 UCL Department of Neuroscience, Physiology and Pharmacology, University College London, Gower Street, London, WC1E 6BT, UK; 2 Neural Plasticity Research Group, Department of Anesthesia & Critical Care, Mass General Hospital & Harvard Medical School, 149 13th Street, Charlestown, MA 02129, USA; 3 Fishberg Department of Neuroscience, Mount Sinai School of Medicine, New York, NY 10029, USA; 4 Pfizer Global Research & Development, Pain Therapeutics, Ramsgate Road, Sandwich, Kent, CT13 9NJ, UK

## Abstract

**Background:**

The role of the neurotrophin regulated polypeptide, VGF, has been investigated in a rat spared injury model of neuropathic pain. This peptide has been shown to be associated with synaptic strengthening and learning in the hippocampus and while it is known that VGFmRNA is upregulated in dorsal root ganglia following peripheral nerve injury, the role of this VGF peptide in neuropathic pain has yet to be investigated.

**Results:**

Prolonged upregulation of VGF mRNA and protein was observed in injured dorsal root ganglion neurons, central terminals and their target dorsal horn neurons. Intrathecal application of TLQP-62, the C-terminal active portion of VGF (5–50 nmol) to naïve rats caused a long-lasting mechanical and cold behavioral allodynia. Direct actions of 50 nM TLQP-62 upon dorsal horn neuron excitability was demonstrated in whole cell patch recordings in spinal cord slices and in receptive field analysis in intact, anesthetized rats where significant actions of VGF were upon spontaneous activity and cold evoked responses.

**Conclusion:**

VGF expression is therefore highly modulated in nociceptive pathways following peripheral nerve injury and can cause dorsal horn cell excitation and behavioral hypersensitivity in naïve animals. Together the results point to a novel and powerful role for VGF in neuropathic pain.

## Background

The spontaneous burning pain, hyperalgesia and allodynia that characterize neuropathic pain are triggered and maintained by a combination of peripheral and central processes [1,2]. Peripheral mechanisms include the onset of ectopic activity in the injured sensory neurons, cross talk between sensory and sympathetic neurons and interaction with peripheral immune cells [3], while central mechanisms include central sensitization through membrane depolarization and homo and heterosynaptic potentiation maintained by loss of inhibition and immune activation [4-6].

At the heart of many of these processes lie the neurotrophins, which in addition to controlling the survival and differentiation of neurons, play a key role in maintaining and modulating the function of adult nociceptive neurons. NGF and BDNF are highly regulated in skin, peripheral and central neurons, and glia following nerve injury and tissue inflammation, and have been repeatedly implicated in the development and maintenance of chronic neuropathic pain states [7-10].

Microarray analysis of dorsal root ganglia (DRG) mRNA following experimental nerve injury has revealed a striking upregulation of another gene regulated by neurotrophins, VGF [11,12]. VGF polypeptide is found in a distinctive restricted cell distribution in the adult brain, peripheral nervous system, and neuroendocrine system, where it is sorted into secretory granules, processed into small peptides by endoproteolytic cleavage, and released upon depolarization [13]. VGF is notable for its rapid and strong regulation by NGF and BDNF, which drive *vgf *gene transcription in vitro and in vivo, increasing VGF mRNA levels up to 50-fold in PC12 cells [13,14]. More recently, VGF regulation has been associated with synaptic strengthening and learning in the hippocampus [15] and to act downstream of BDNF to increase cell division in the hippocampus and counteract depression in animal models [16]. Despite the reported regulation of VGF mRNA by peripheral nerve injury, it is not known if VGF plays a role in neuropathic pain.

Here we report that VGF is upregulated in both DRG and dorsal horn neurons in a model of neuropathic pain and show that VGF peptide application to the naïve spinal cord directly influences dorsal horn neuron excitability and induces typical neuropathic behavior.

## Results

### Pattern of VGF mRNA and protein upregulation in DRG neurons following spared nerve injury

**Figure 1:**
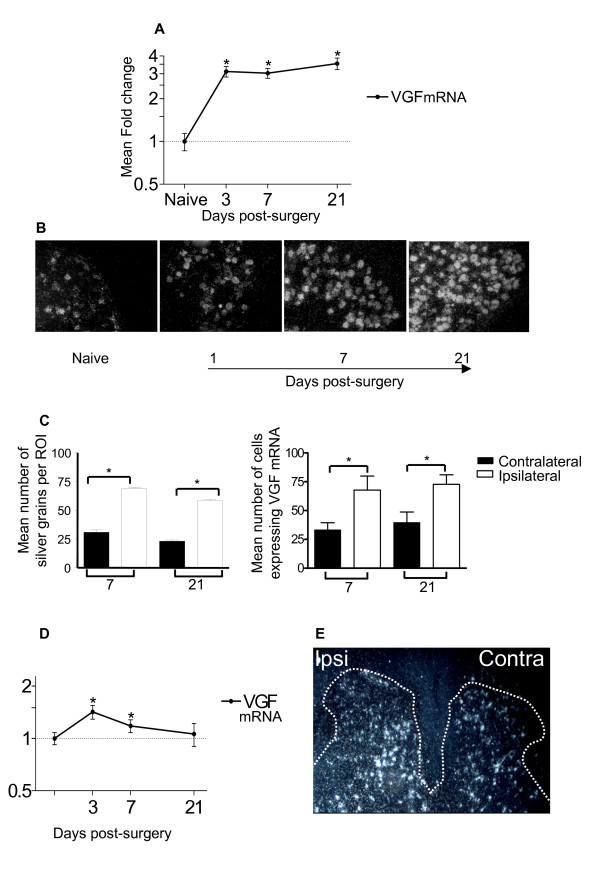
**Increased VGF mRNA expression in L4/5 DRG and dorsal horn following SNI**. A. QPCR analysis of VGF mRNA in ipsilateral L4/5 DRG from naïve and 3, 7 and 21 days post-SNI. Data is expressed as the mean fold change ± standard deviation normalised to naïve values (* *p *≤ 0.05, ANOVA, n = 8 per group). B In situ hybridization histochemistry of L4/5 DRG showing the change in VGF mRNA expression post-injury. C Quantification of in situ hybridization data showing the mean number of ipsilateral and contralateral DRG cells expressing VGF mRNA and the mean density of silver grains at 7 and 21 days post-SNI (* *p *≤ 0.05, *ANOVA*, Dunnetts post-hoc test, n = 4 per group). D. QPCR analysis of VGF mRNA from L4/5 naïve and ipsilateral dorsal horn quadrants, 3, 7 and 21 days post-SNI. Data is expressed as the mean fold change ± standard deviation and normalised to naïve values (*p ≤ 0.05, ANOVA, n = 8 per group). E. In situ hybridization of VGF mRNA in L4/5 spinal cord at 7 days post-SNI.

We used the hindlimb spared nerve injury (SNI) model of neuropathic pain to examine the dynamic regulation of VGF in primary sensory neurons in the rat L4/L5 dorsal root ganglia (DRG). Figure 1A shows that SNI triggers a sustained 3 fold upregulation of VGF mRNA that is maintained for at least 3 weeks. In situ hybridization (Figure 1B) shows that this upregulation is restricted to DRG neurons; no VGF mRNA expression is observed in glial or satellite cells. The number of neurons expressing VGF mRNA increases steadily post injury with a time course that parallels the qPCR data. Quantification of the *in situ *hybridization shows that both the amount of VGF mRNA per cell and the number of cells expressing VGF mRNA is significantly greater in the ipsilateral compared to the contralateral DRG at 7 and 21 days post injury (Figure 1C & D).

**Figure 2:**
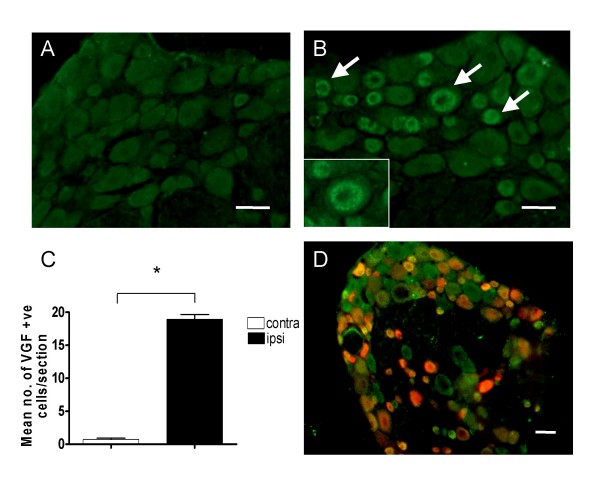
**Increased VGF protein expression in L4/5 DRG following SNI**. A. VGF protein immunostaining in naive L4 DRG shows very low basal levels compared to B. 7 days following SNI where a significant increase in the expression of VGF in all diameter cell bodies can be seen. Inset shows detail of punctuate cytoplasmic VGF staining. C. The mean number of cells/section expressing VGF in the ipsilateral and contralateral L4 DRG, 21 days post SNI highlights this increase in ipsilateral labeling for VGF (* *p *≤ 0.05, t-test, n = 3 per group). D. Retrograde labelling of injured cell bodies (red) shows VGF expression is largely found in both large and small injured cell bodies (orange, arrows), although expression is also visible in uninjured/untraced DRG cell bodies (arrow). Scale bars 50 μm.

VGF protein is also upregulated in the L4 and L5 DRG following SNI. Figure 2A shows the low level of VGF immunoreactivity in naïve L4 DRG compared to the ipsilateral L4 DRG 7 days following SNI in figure 2B. Increased VGF expression was observed in both large and small diameter DRG neurons and staining was cytoplasmic and punctate in nature, consistent with its storage in secretory vesicles (Levi et al., 2004). Figure 2C shows that by day 21 post injury there is a 20 fold increase in VGF positive cells in the ipsilateral relative to contralateral DRG. To establish whether the VGF upregulation was in neurons whose axons were damaged by SNI surgery, we applied a retrograde tracer (True blue) to the cut end of the peripheral nerves at the time of the injury. Figure 2D shows that increased VGF expression in ipsilateral L4 DRG 7 days post injury is largely, but not entirely restricted to injured neurons.

Taken together these data clearly show strong upregulation of VGF mRNA and protein in injured DRG neurons following nerve injury.

### Pattern of VGF mRNA and protein upregulation in dorsal horn neurons following spared nerve injury

VGF mRNA and protein is also increased in the L4/L5 dorsal horn after SNI. Figure 1D shows the significant mean fold increase in mRNA in the ipsilateral L4/5 dorsal horn at 3 and 7 days post injury. In situ hybridization revealed that the pattern of mRNA upregulation is not uniform across the dorsal horn. It appears that mRNA increased in neurons in the superficial laminae and in deeper lamina IV-V, while neurons in inner lamina II-III remained relatively unaffected (Figure 1E) although this requires further quantitation.

**Figure 3:**
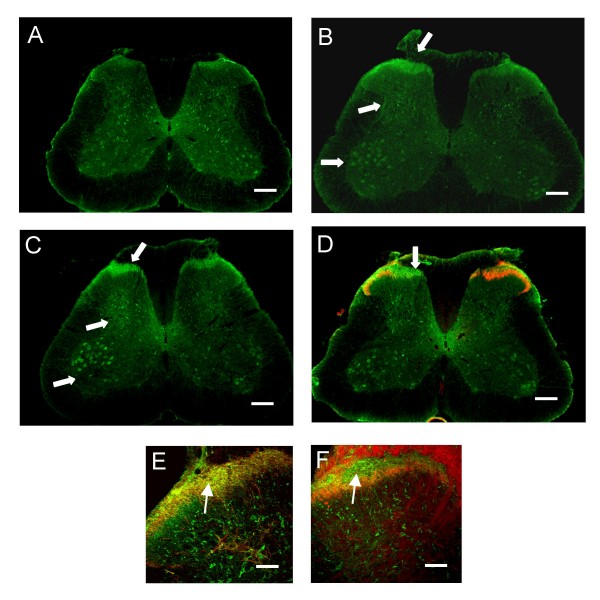
Immunohistochemistry of VGF in the spinal cord of (A) naïve and (B) nerve injured rats at 7 days and (C) 21 days post-SNI. Arrows show increased VGF staining in the C fibre terminal region of the ipsilateral superficial laminae (LI-II), intrinsic dorsal horn neurons throughout the dorsal horn and ventral horn neurons (n = 4 per group). D. Increased VGF expression in the central afferent terminals (arrow) coincides with afferent IB4 depletion following SNI. (Scale bars: 100 μm). E. Double immunolabeling of naïve (uninjured) spinal cord sections shows colocalisation in terminals (yellow, arrow) of VGF (green) and CGRP (red) a marker of peptidergic C-fibres. F. Double immunolabeling of naïve uninjured spinal cord sections shows little overlap in VGF (green, arrow) and IB4 (red), a marker of non-peptidergic C-fibres (Scale bars: 50 μm).

A similar pattern of upregulation of VGF protein expression is observed in the dorsal horn (Figure 3A–D). In the naïve L4 spinal cord (Figure 3A), both neurons and terminals are immunolabelled with VGF. Neuronal VGF is largely restricted to scattered neurons in laminae III-IV and the ventral horn and light terminal labeling is observed around the central canal and lamina I and II. These terminals partly arise from primary afferents as shown by the decrease following dorsal rhizotomy and partly from descending axons in the dorsolateral funiculus (DLF) as shown by DLF lesions and co-labelling of back-labelled descending projection neurones and VGF in the brainstem (Additional file 1). Some VGF staining is likely also to arise from the terminals of spinal interneurons. The VGF primary afferent terminal labeling overlaps with CGRP+ve (Figure 3E) but not IB4+ve (Figure 3F) primary afferents in the superficial dorsal horn.

Seven and 21 days after SNI there is a substantial increase in terminal labeling in superficial laminae and a more diffuse upregulation in terminals in laminae III-V (Figure 3B & C). This increased terminal labeling is restricted to the somatotopic region of termination of the affected afferents in the L4 and L5 segments (Figure 3D). In addition, VGF protein is upregulated in intrinsic neurons in both superficial and deep laminae following nerve injury. The number of VGF-labelled cells in laminae II-V on the ipsilateral side was more than 3 times greater than the contralateral side at 7 days post injury (ipsi: 21.8 ± 1.6, contra: 6.6 ± 0.6 cells/section, n = 14 sections from 4 animals). Double labeling with specific neuronal, microglial and astrocytic markers confirmed that the VGF protein in post injury dorsal horn was restricted to neurons (Additional file 2).

Taken together these data show strong and selective upregulation of VGF mRNA and protein in dorsal horn neurons and terminals following nerve injury.

### Spinal VGF application causes behavioral mechanical and cold hypersensitivity

Since VGF is highly regulated in the DRG and dorsal horn following peripheral nerve injury, we hypothesized that it may have a role in the behavioral hypersensitivity to touch and cold that is a characteristic of neuropathic pain. We therefore investigated the effects of acute intrathecal application of TLQP-62 (the C-terminal 62 amino acid VGF-derived peptide) upon hindlimb flexion withdrawal in response to the application of acetone and to graded mechanical stimulation of the hindpaw.

**Figure 4:**
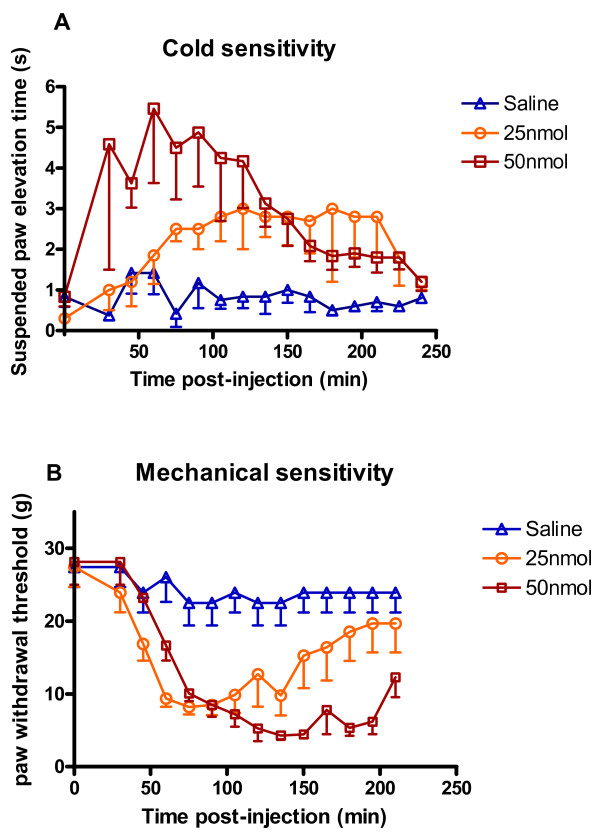
The effects of intrathecal TLQP-62 and saline vehicle on reflex withdrawal responses to innocuous cold and mechanical stimuli in naive rats. A. Mean suspended paw elevation time in seconds to acetone applied to the hind paw B. Mean paw withdrawal threshold (g) to mechanical von Frey hair stimulation after intrathecal administration of TLQP-62. Each point is the mean of 8 rats.

Figure 4A shows the increased paw sensitivity to cutaneous acetone cooling following L4/5 intrathecal application of 25 and 50 nmol TLQP-62. This is an innocuous cooling stimulus that causes very little response in control animals but both 25 and 50 nmol TLQP-62 cause a significant increase in the paw withdrawal response to cooling compared to saline (P < 0.001, ANOVA). The effect is prolonged, a single intrathecal injection lasting for nearly 2 hours. A dose response relationship can be observed in the Fig 4A, although this was not significant.

Fig 4B shows the increased paw sensitivity to cutaneous mechanical stimulation with von Frey hairs following 25 and 50 nmol TLQP-62. Both doses cause a significant decrease in the paw withdrawal thresholds compared to saline (P < 0.05 and 0.001, ANOVA). Again the effect is prolonged, the response to a single intrathecal injection lasting for 3 hours. A dose response trend in the duration of the effect can be observed in Fig 3B, although this was not significant.

Together these data show that VGF, applied directly to the spinal cord, causes comparable mechanical and cooling hypersensitivity in naïve animals to that reported following nerve injury.

### VGF influences the excitability of dorsal horn neurons

**Figure 5:**
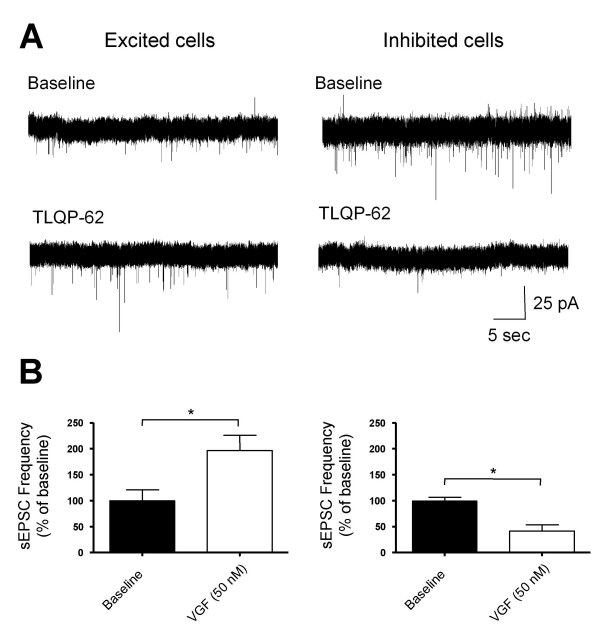
TLQP-62 alters the spontaneous excitability of superficial dorsal horn neurones. sEPSCs were recorded in lamina I and II neurones from P21 spinal cord slices using whole cell patch clamp techniques. Cells were clearly divided in their response to TLQP-62 (50 nM) with 6/9 neurones showing a significant increase in the frequency of sEPSCs while 3/9 cells were significantly inhibited, as illustrated by sample traces (A) and histograms showing the normalised frequency of spontaneous activity before and 10 minutes after TLQP-62 application (B). The effects were classified as inhibitory or excitatory using a Komologorov-Smirnov 2-sample test (P < 0.05) and a t-test was used to compare the population data in B (*P < 0.05).

To test whether the behavioural hypersensivity following intrathecal VGF is due to changes in dorsal horn neuronal excitability we investigated the effects of acute application of the C-terminal VGF-derived peptide TLQP-62 upon the activity of single superficial dorsal horn neurons in rat spinal cord slices from naïve animals. The frequency of spontaneous excitatory post synaptic currents (sEPSC) was significantly altered by bath application of 50 nM TLQP-62 in the majority (82%, n = 11) of cells examined (Figure 5). Of these cells most (70%) were excited, with a 2-fold increase in the mean sEPSC frequency (Figure 5B). A proportion of cells (30%) were inhibited by TLQP-62 and displayed a 50% decrease in sEPSC frequency (Figure 5B). The peak effect of TLQP-62 was observed after 5–10 minutes of application and in all but one cell, firing had returned to baseline levels after 30 minutes of wash out in normal aCSF

**Figure 6:**
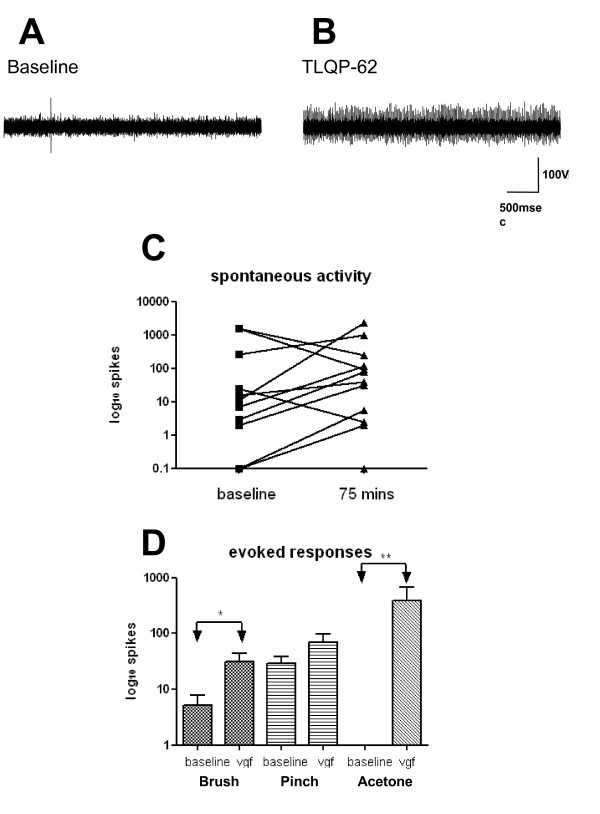
The effect of spinal TLQP-62 (50 nM) application upon L4/L5 dorsal horn cell activity in intact anaesthetized rats. A & B. A dorsal horn cell that had very little spontaneous spike activity at baseline, developed very high frequency spontaneous firing, shown here 50 mins post TLQP-62. C. The effect of spinal TLQP-62 application upon spontaneous activity in individual dorsal horn cells. The number of action potentials over a 10 min period is shown at baseline and at 75 mins post TLQP-62. The logarithmic scale reflects the very intense effect of TLQP-62 in a subpopulation of cells. D. The effect of spinal TLQP-62 upon brush, pinch and acetone evoked activity in dorsal horn cells (n = 14). The mean number of action potentials evoked by each stimulus (duration 1.5 secs) is shown at baseline and at 75 mins post TLQP-62. *P < 0.05, **P < 0.001 ; Mann-Whitney U test).

The effect of VGF peptide was also tested on dorsal horn cells *in vivo *in naïve isoflurane anaesthetized rats in prolonged extracellular recordings of single wide dynamic range neurons in laminae III-V (Figure 6). Neuronal activity was analysed every ten minutes before and for up to 100 minutes after application of TLQP-62 (50 nM). In the majority of neurons (77%, n = 14) spontaneous firing significantly increased and in a subpopulation of cells (n = 4) strikingly so (Figure 6A & B). In a smaller population of cells (15%) the spontaneous firing was decreased and the remainder was unchanged. The effect of TLQP-62 was also tested on the activity evoked by stimulating cutaneous receptive fields (Figure 6C). Application of TLPQ-62 significantly increased the mean response to brushing and cooling the receptive field while leaving pinch responses unaffected. The most striking effect was to acetone application, which in this cell population had no effect at baseline. Application of TLQP-62 led to a strong cooling response in 70% of cells and again the effect was intense in a subpopulation (n = 4) (Figure 6C).

These results show that VGF can directly influence spontaneous and evoked responses of activity of naïve dorsal horn cells.

## Discussion

The results show that the polypeptide VGF is strongly upregulated in injured DRG neurons and their central target dorsal horn neurons following SNI.

The onset and maintenance of this upregulation mirrors the time course of behavioral allodynia that is a feature of this animal model of neuropathic pain [17]. The baseline distribution and cellular location of VGF peptide in neurons, but not glial cells is consistent with earlier studies describing the distribution of this peptide in the brain [18,19,13]. Identification of VGF receptors will allow further insight into its neuronal and glial targets.

The striking upregulation of VGF in sensory neurons following nerve injury is reminiscent of the selective and maintained activation of VGF in other brain regions in response to neurotropins and prolonged neuronal depolarization [13,15,14] and is likely to result from injury induced spike activity and neurotrophin upregulation in the DRG and dorsal horn. Peripheral nerve damage triggers NGF release by macrophages, mast cells and Schwann cells, [10] and BDNF upregulation in trkA expressing DRG cells. Anti-NGF treatment leads to reduction of allodynia in peripheral nerve injury models [10] and many of the injury induced changes in dorsal horn neuronal excitability are mediated by release of BDNF from microglia [7]. It is possible that these effects are mediated via VGF or select VGF-derived peptides. The colocalization of VGF with peptidergic primary afferents, in agreement with previous reports in culture [10], suggests depolarisation and neurotrophin mediated VGF release from a select group of nociceptors.

VGF application in naive animals had striking behavioural effects that mirror those of neuropathic pain; an allodynic response to cooling and mechanical stimulation are major features of this type of chronic pain [1]. Our results show that VGF acts, at least in part, by selective changes in the excitability of subpopulations of dorsal horn cells. While mixed responses are likely to reflect the presence of both excitatory and inhibitory neurons, the overall effect of VGF peptide was to increase global dorsal horn network activity, analogous to BDNF [20]. This was supported by the in vivo receptive field analysis which showed that VGF peptide significantly increased spontaneous activity and evoked responses to cooling.

## Conclusion

In conclusion we identify a neuropeptide that has profound functional effects on dorsal horn neurons and sensory behavior. Taken together with its upregulation in primary sensory and dorsal horn neurons in response to peripheral nerve injury, these data suggest that VGF has a central mechanistic role in neuropathic hypersensitivity. Such a role can only be fully established following identification of a receptor and effective blockade of VGF function in sensory pathways.

## Methods

All animal procedures were carried out on Sprague Dawley rats and performed in accordance with the Animals (Scientific Procedures) Act 1986.

*Spared Nerve Injury (SNI) *was carried out in adult rats under 2–3% halothane/O_2 _anesthesia [17]. The tibial and common peroneal branches of the sciatic nerve were tightly ligated with 5/0 silk and axotomised, leaving only the sural nerve intact. Cut ends of the sciatic nerve were dipped in 2% True Blue (Sigma) for 3 min and left for 7–21 days for retrograde labeling.

*In situ hybridization *was performed on frozen transverse sections of spinal cord (10 μm) as described previously [21] using complementary and specific rat VGF mRNA oligonucleotides (SigmaGenosys, UK). The oligonucleotides were 3' end-labeled with deoxyadenosine % Warning: EPS-printer was not specified – α [^35^S]-triphosphate (specific activity <1250 Ci/mol; DuPont NEN) using terminal deoxynucleotidyl transferase (Promega, UK). Specificity controls were (1) pretreating sections with RNase A (1 mg/ml; Sigma, Poole, UK) for 1 hr before hybridization and (2) coincubation of the^35^S-labeled oligonucleotide in the hybridization medium with a 100-fold excess of unlabeled oligonucleotide.

*Immunohistochemical staining *was performed on 40 μm free-floating cryosections of L4/L5 spinal cord. For VGF, the sections were blocked for 1 hr in TTBS (0.05 M Tris saline, pH7.4/0.3% Triton-X100) containing 3% normal rabbit serum (NRS) at room temperature and incubated at 4°C for 72 hr with goat α-VGF (G17, Santa Cruz, CA) diluted 1:5000 in TTBS. Followed after three 10 min washes in 0.1 M phosphate buffer by a 90 min incubation at room temperature with biotinylated rabbit anti-goat secondary antibody (Vector Laboratories, Burlingame, CA) diluted 1:200 in TTBS. A further three washes in 0.1 M phosphate buffer were followed by a 60 min incubation at room temperature with Avidin Biotin complex (2.5 μl A, 2.5 μl B per ml of TTBS). Signal was amplified using a tyramide amplification protocol. Controls were carried out without the primary antibody. In addition, 3 independent primary antibodies to VGF were used. VGF (R15) which recognises the C-terminus of VGF, VGF (D20) which recognises the N-terminal of VGF and VGF (G17) which also recognises the N-terminus. All three antibodies showed the same clear and consistent increase in staining in the DRG and dorsal horn following SNI despite being raised to three separate VGF epitopes. Ultimately, VGF (G17) was selected as the staining was the clearest.

Sections were double labeled for neuronal nuclei (mouse anti-NeuN antibody (Chemicon, CA) 1:5000), calcitonin gene regulated polypeptide (rabbit anti CGRP antibody (Chemicon, CA) 1:4000), ionized binding calcium adaptor molecule-1 (rabbit anti-iba-1 (Wako, Japan) 1:2000), isolectin B4 ((Sigma) 1:200), 5 HT (monoclonal rat anti-serotonin, (Chemicon, CA)1:75).

*Quantitative real-time PCR (RT-PCR) *was performed using the SYBR green detection system with primer sets designed on Primer Express (Applied Biosystems, UK). Specific PCR product amplification was confirmed using dissociation protocol. Transcript regulation was determined using the relative standard curve method per manufacturers' instructions. Relative loading was determined prior to RT-PCR with RNA spectrophotometry followed by gel electrophoresis and post RT-PCR by amplification of glyceraldehyde-3-phosphate dehydrogenase (GAPDH). For each time point 4 samples of pooled tissue from 2 rats were analyzed.

### Behavioural testing

Flexion withdrawal reflex thresholds to punctate mechanical stimulation of the plantar surface of the hindpaw were established in naïve adult rats using von Frey filaments (VF) (Stoelting, Woodvale, Il) applied sequentially to the plantar surface of the hind paw 10 times at one second intervals. Threshold was defined as the VF filament causing paw 50% withdrawal. Cold responses were measured by placing a drop of acetone on the plantar of the paw and recording the number of seconds that the paw was withdrawn over the following 20 seconds. Rats were tested one day prior to intrathecal injections to establish baseline sensitivity and again immediately before anaesthetising for intrathecal injection with TLQP-62 (C-terminal 62 amino acid rat VGF-derived peptide) or vehicle. Mechanical and cold sensitivity were then tested 30 min after application and every 15 minutes for four hours Intrathecal (10 μl of drug or saline) injections were performed at the L4–5 level in anesthetized rats (2–3 % halothane/O_2_) using a 10 μl 26 G Hamilton syringe. The experimenter was blind to the contents of the syringe and only one sensory modality was tested per rat.

*Single unit extracellular electrophysiological recordings *were carried out in the L4–5 dorsal horn of naïve adult rats using standard in vivo recording techniques [22]. Rats were anesthetized with isoflurane (4% in O_2 _at 400 ml/min for induction, 2.5% at 200 ml/min for maintenance). Single spikes with cutaneous receptive fields on the hindpaw were recorded throughout the depth of the dorsal horn and analysed using Chart software (AD Instruments, UK). Spontaneous activity was recorded for one minute followed by 3 brush stimuli and 3 pinch stimuli (1.5 sec duration each at 30 secs intervals). A single drop of acetone was the applied to the receptive field and responses recorded for 30 seconds. Following baseline recording, 20 ul of TLQP-62 (50 nM) in saline was applied directly onto the exposed spinal cord and stimuli were repeated at 5 and 10 minutes post drug application and every ten minutes thereafter for 60–120 minutes.

### Whole cell patch recording in spinal cord slices

Superficial dorsal horn neurons were patched at room temperature in 400 μm lumbar sagittal slices in 21 day old naïve rats [23]. Pipette resistances ranged from 3–5 MΩ, seal resistances were > 1 GΩ and electrodes were filled with (in mM): 130 K-gluconate, 10 KCl, 0.1 CaCl_2_,1 EGTA, 10 HEPES, 2 MgATP (pH 7.2, 300–305 mosm). Superficial dorsal horn neurons were visualized with IR-DIC, and voltage clamp recordings of whole-cell currents were obtained using a Multiclamp 700A amplifier (Axon Instruments, CA). Spontaneous excitatory post-synaptic potentials (sEPSCs) were observed at a holding potential of -70 mV and baseline activity was established for at least 5 min before drug application. TLQP62 (50 nM in aCSF) was bath applied for up to 30 minutes and its effects on sEPSC amplitude and frequency analysed off-line.

## List of abbreviations

BDNF: brain derived neurotrophic factor; CGRP: calcitonin gene related peptide; DLF: dorsal lateral funiculus; DRG: dorsal root ganglia; NGF: nerve growth factor; sEPSC spontaneous excitatory postsynaptic potential; SNI: spared nerve injury.

## Competing interests

The authors declare that they have no competing interests.

## Authors' contributions

AM conceived and performed behavioural, histological and quantitiative PCR studies as well as interpreting data and aiding in the preparation of the manuscript. RI conceived and performed synaptic physiology experiments. SK performed *in vivo *physiological studies. AT performed immunohistochemical studies. LL performed *in situ *hybridization studies. MB conceived and performed some synaptic physiology experiments. GH contributed to the behavioural investigations as well as preparation of the manuscript. MC performed qPCR and behavioral investigations. SS discovered and provided VGF for this study and aided in the conception of this work. MF conceived the project, lead the experimental design and data analysis and prepared the final manuscript.

## Supplementary Material

Additional file 1 Origins of terminal staining of VGF in the lumbar spinal cord. A. Immunohistochemistry of VGF (left) and CGRP (right) in the L4 spinal cord following unilateral L3–5 dorsal rhizotomy 5 days earlier. Ipsilateral CGRP depletion indicates total loss of primary afferent input. VGF staining in the region is decreased but not totally depleted (arrow). B. VGF (left) and 5 HT (right) immunostaining in L4 spinal cord following unilateral lesion of the dorsolateral funiculus at upper thoracic level 5 days earlier. Ipsilateral 5-HT depletion indicates loss of descending brainstem terminals, while VGF is only partially diminished (arrow). C Retrograde labeling of projection neurones in the rostroventral medulla, using bilateral True blue (2%) injection into the L4/5 spinal cord under anaesthetic, 5 days earlier (red), demonstrates expression of VGF in brainstem descending projection neurones. True blue:red. VGF:green. Double labeled: orange. Click here for file

Additional file 2 Origins of intrinisic staining of VGF in the lumbar spinal cord. A Immunohistochemistry for VGF and NeuN highlighting the ipsilateral increase in VGF in intrinsic dorsal horn neurones (Arrow). B High-power confocal immunohistochemistry of VGF and markers for microglia (Iba-1), neurones (NeuN) and astrocytes (GFAP) demonstrates that intrinsic VGF protein expression is co-localised specifically with the neuronal marker NeuN but not with either of the glial markers (arrows). Click here for file
