# Supplementary material for: Origins, actions and dynamic expression patterns of the neuropeptide VGF in rat peripheral and central sensory neurones following peripheral nerve injury
Source: Mol Pain. 2008 Dec 10;4:62. doi: 10.1186/1744-8069-4-62 (PMC2614976; doi:10.1186/1744-8069-4-62)
Supplement: Additional file 1 — Origins of terminal staining of VGF in the lumbar spinal cord. A. Immunohistochemistry of VGF (left) and CGRP (right) in the L4 spinal cord following unilateral L3–5 dorsal rhizotomy 5 days earlier. Ipsilateral CGRP depletion indicates total loss of primary afferent input. VGF staining in the region is decreased but not totally depleted (arrow). B. VGF (left) and 5 HT (right) immunostaining in L4 spinal cord following unilateral lesion of the dorsolateral funiculus at upper thoracic level 5 days earlier. Ipsilateral 5-HT depletion indicates loss of descending brainstem terminals, while VGF is only partially diminished (arrow). C Retrograde labeling of projection neurones in the rostroventral medulla, using bilateral True blue (2%) injection into the L4/5 spinal cord under anaesthetic, 5 days earlier (red), demonstrates expression of VGF in brainstem descending projection neurones. True blue:red. VGF:green. Double labeled: orange. [file 1744-8069-4-62-S1.ppt]

## Slide 1
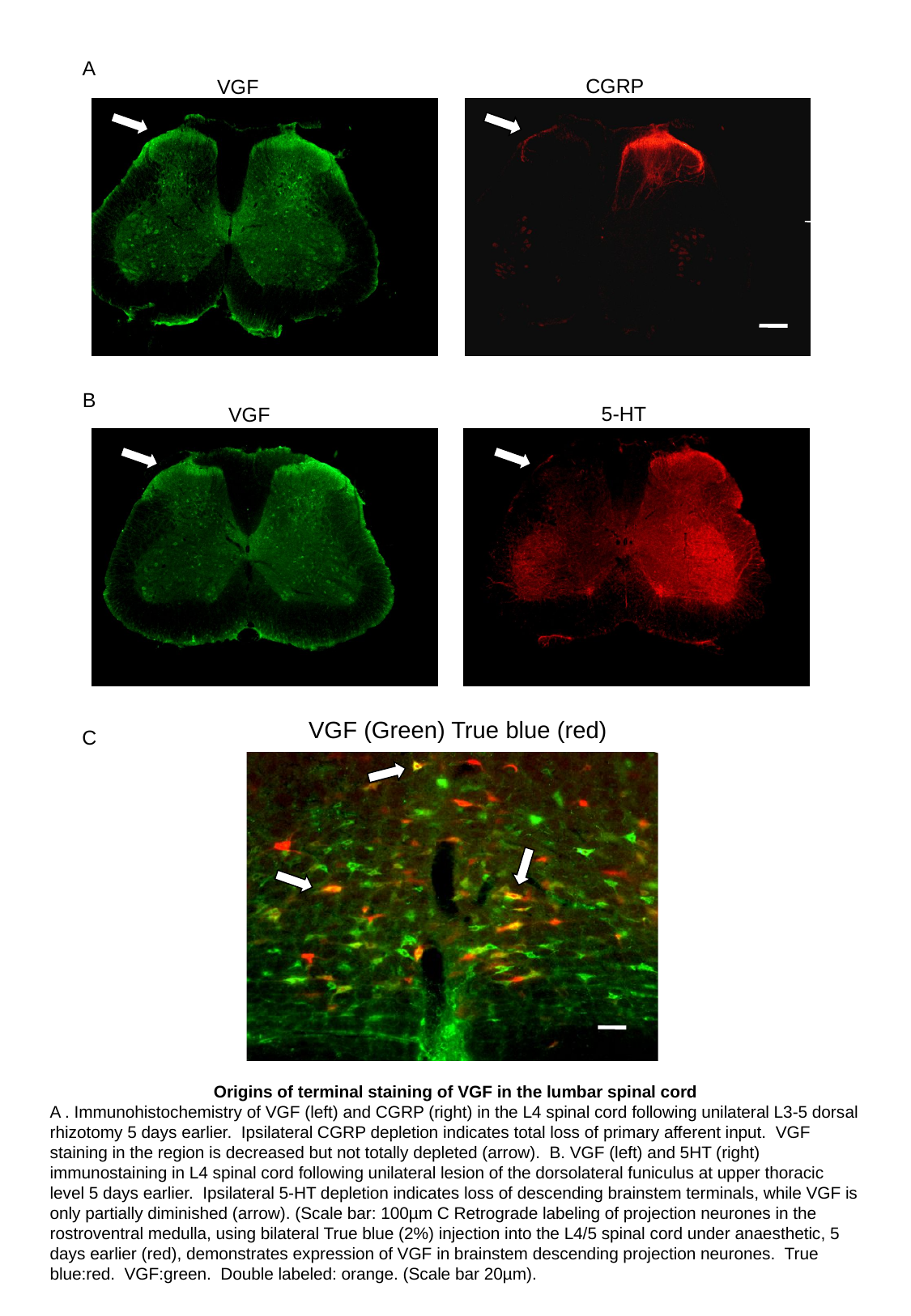

C
I
C
I
C
A
CGRP
VGF
I
C
B
5-HT
VGF
VGF (Green) True blue (red)
C
Origins of terminal staining of VGF in the lumbar spinal cord
A . Immunohistochemistry of VGF (left) and CGRP (right) in the L4 spinal cord following unilateral L3-5 dorsal rhizotomy 5 days earlier. Ipsilateral CGRP depletion indicates total loss of primary afferent input. VGF staining in the region is decreased but not totally depleted (arrow). B. VGF (left) and 5HT (right) immunostaining in L4 spinal cord following unilateral lesion of the dorsolateral funiculus at upper thoracic level 5 days earlier. Ipsilateral 5-HT depletion indicates loss of descending brainstem terminals, while VGF is only partially diminished (arrow). (Scale bar: 100µm C Retrograde labeling of projection neurones in the rostroventral medulla, using bilateral True blue (2%) injection into the L4/5 spinal cord under anaesthetic, 5 days earlier (red), demonstrates expression of VGF in brainstem descending projection neurones. True blue:red. VGF:green. Double labeled: orange. (Scale bar 20µm).
