# Supplementary material for: Origins, actions and dynamic expression patterns of the neuropeptide VGF in rat peripheral and central sensory neurones following peripheral nerve injury
Source: Mol Pain. 2008 Dec 10;4:62. doi: 10.1186/1744-8069-4-62 (PMC2614976; doi:10.1186/1744-8069-4-62)
Supplement: Additional file 2 — Origins of intrinisic staining of VGF in the lumbar spinal cord. A Immunohistochemistry for VGF and NeuN highlighting the ipsilateral increase in VGF in intrinsic dorsal horn neurones (Arrow). B High-power confocal immunohistochemistry of VGF and markers for microglia (Iba-1), neurones (NeuN) and astrocytes (GFAP) demonstrates that intrinsic VGF protein expression is co-localised specifically with the neuronal marker NeuN but not with either of the glial markers (arrows). [file 1744-8069-4-62-S2.ppt]

## Slide 1
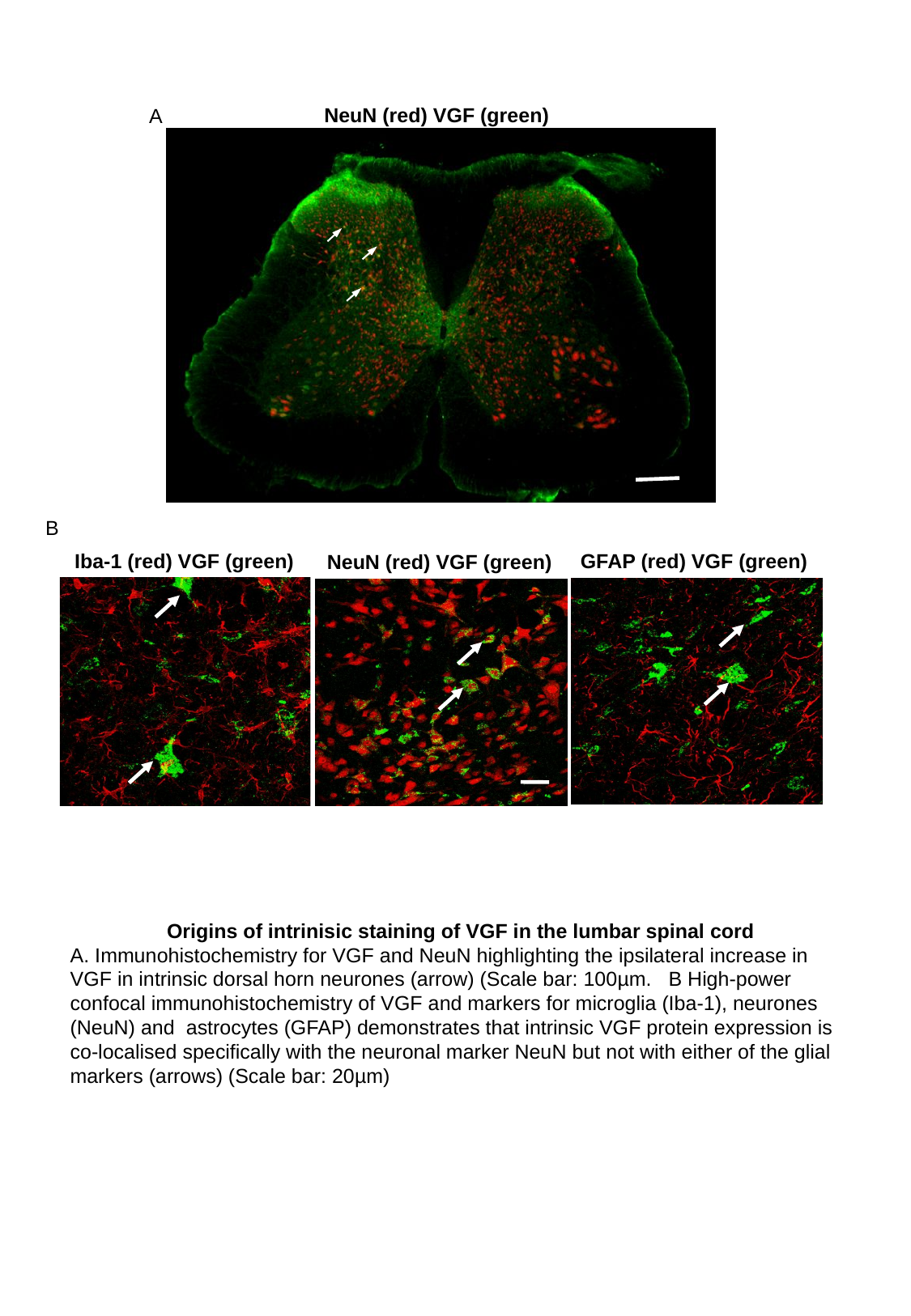

I
C
I
C
I
C
NeuN (red) VGF (green)
A
B
Iba-1 (red) VGF (green)
GFAP (red) VGF (green)
NeuN (red) VGF (green)
Origins of intrinisic staining of VGF in the lumbar spinal cord
A. Immunohistochemistry for VGF and NeuN highlighting the ipsilateral increase in VGF in intrinsic dorsal horn neurones (arrow) (Scale bar: 100µm. B High-power confocal immunohistochemistry of VGF and markers for microglia (Iba-1), neurones (NeuN) and astrocytes (GFAP) demonstrates that intrinsic VGF protein expression is co-localised specifically with the neuronal marker NeuN but not with either of the glial markers (arrows) (Scale bar: 20µm)
